# Green turtles shape the seascape through grazing patch formation around habitat features: Experimental evidence

**DOI:** 10.1002/ecy.3902

**Published:** 2022-12-21

**Authors:** F. O. H. Smulders, E. S. Bakker, O. R. O'Shea, J. E. Campbell, O. K. Rhoades, M. J. A. Christianen

**Affiliations:** ^1^ Aquatic Ecology and Water Quality Management Group Wageningen University & Research Wageningen The Netherlands; ^2^ Department of Aquatic Ecology Netherlands Institute of Ecology (NIOO‐KNAW) Wageningen The Netherlands; ^3^ Wildlife Ecology and Conservation Group Wageningen University Wageningen The Netherlands; ^4^ The Centre for Ocean Research and Education (CORE) Gregory Town The Bahamas; ^5^ Department of Biological Sciences Institute of Environment, Florida International University Miami Florida USA

**Keywords:** *Chelonia mydas*, habitat structure, herbivory, landscape of fear, plant–herbivore interactions, seagrass, *Thalassia testudinum*

## Abstract

Understanding how megaherbivores incorporate habitat features into their foraging behavior is key toward understanding how herbivores shape the surrounding landscape. While the role of habitat structure has been studied within the context of predator–prey dynamics and grazing behavior in terrestrial systems, there is a limited understanding of how structure influences megaherbivore grazing in marine ecosystems. To investigate the response of megaherbivores (green turtles) to habitat features, we experimentally introduced structure at two spatial scales in a shallow seagrass meadow in The Bahamas. Turtle density increased 50‐fold (to 311 turtles ha^−1^) in response to the structures, and turtles were mainly grazing and resting (low vigilance behavior). This resulted in a grazing patch exceeding the size of the experimental setup (242 m^2^), with reduced seagrass shoot density and aboveground biomass. After structure removal, turtle density decreased and vigilance increased (more browsing and shorter surfacing times), while seagrass within the patch partly recovered. Even at a small scale (9 m^2^), artificial structures altered turtle grazing behavior, resulting in grazing patches in 60% of the plots. Our results demonstrate that marine megaherbivores select habitat features as foraging sites, likely to be a predator refuge, resulting in heterogeneity in seagrass bed structure at the landscape scale.

## INTRODUCTION

The physical arrangement of objects in space can determine the movements and grazing behavior of large herbivores (Treydte et al., 2010). Habitat structure may be used as shelter, for orientation, or as a food source, locally increasing the grazing impact and therefore shaping the surrounding landscape (Anderson et al., 2010; Khadka & James, 2016). Habitat structure also plays an important role in predator–prey dynamics (Owen‐Smith, 2019). For example, herbivore prey that are chased down by predators are likely to use habitat features as both a place for shelter as well as to forage, indicated by locally increased grazing and reduced plant biomass (Bakker et al., 2005; Creel et al., 2005). Conversely, ambush predators may incorporate habitat features such as tree logs or rocky outcrops in their hunting strategy (Podgórski et al., 2008; Smith et al., 2019), causing prey to avoid grazing near these features (van Ginkel et al., 2019). In contrast with terrestrial ecosystems, there is a limited understanding of how habitat features influence megaherbivore grazing behavior in marine ecosystems.

Seagrass ecosystems provide important foraging habitat for large marine grazers, in which patch reefs (e.g., coral boulders) as well as man‐made structures (e.g., jetties or wrecks) commonly occur and provide some habitat structure. Green turtles (*Chelonia mydas*) use these shallow seagrass systems as foraging grounds and display high foraging site fidelity (Shimada et al., 2020). Anecdotal evidence describes that turtles may use vertical habitat features as refuge from predation from sharks (Thomson et al., 2011), and that turtles use coral boulders or caves to rest in at night, probably as shelter from predation (Christiansen et al., 2017). Additionally, a preference for safer edge habitats instead of interior shallow banks has been described for turtles in Shark Bay, Australia, with impacts on grazing behavior and vegetation structure (Burkholder et al., 2013; Heithaus et al., 2007). This can be explained by the hunting preference of the main turtle predators, tiger sharks (*Galeocerdo cuvier*), for homogeneous shallow seagrass meadows where escape opportunities for prey are limited (Heithaus, Dill, et al., 2002; Heithaus, Frid, & Dill, 2002). Small marine herbivores such as urchins and fishes use structural features such as coral formations as shelter, increasing grazing pressure around these structures and forming grazing “halos” (DiFiore et al., 2019), however experimental evidence of a similar mechanism for larger marine herbivores is lacking. Therefore, it remains unknown whether large marine grazers, such as turtles, incorporate natural or artificial habitat features in their foraging site selection and, if so, what the specific requirements (e.g., dimensions, number of features) are of those habitat features.

In this study, we assessed if and how green turtles exhibit variation in grazing behavior and impact in response to the presence of habitat features. Additionally, we explore whether this behavior is dependent on the size of an area with habitat features. To this purpose, we experimentally added artificial structures (mesh cages) in both large‐scale and small‐scale arrays to a shallow bay with extensive seagrass meadows on Eleuthera, The Bahamas. Based on previous findings that turtles seek shelter near corals at night, as well as their known predator–prey dynamics, we expect turtles to select structures in both the small‐ and large‐scale arrays as their preferred foraging site, resulting in local increases in turtle density, a decrease in vigilant behavior and grazing patch initiation with impacts on seagrass structure.

## MATERIALS AND METHODS

### Study site

The experiments were conducted at Bottom Harbour, north Eleuthera, The Bahamas (25.465294, −76.634903) from May 2018 to August 2020. Bottom Harbour is a shallow water inlet of the western Atlantic Ocean with a mean depth of ~3.5 m, dominated by a continuous high‐cover *Thalassia testudinum* seagrass meadow. The bay provides a year‐round foraging site for subadult green turtles (*C. mydas*), and is situated within The Bahamas shark sanctuary (Gallagher et al., 2021). Large numbers of turtle predators (tiger shark, *G. cuvier*) have been reported in the region of our study site (Talwar et al., 2020 and others, summarized in Appendix S1: Table S1).

### Experimental design

**FIGURE 1:**
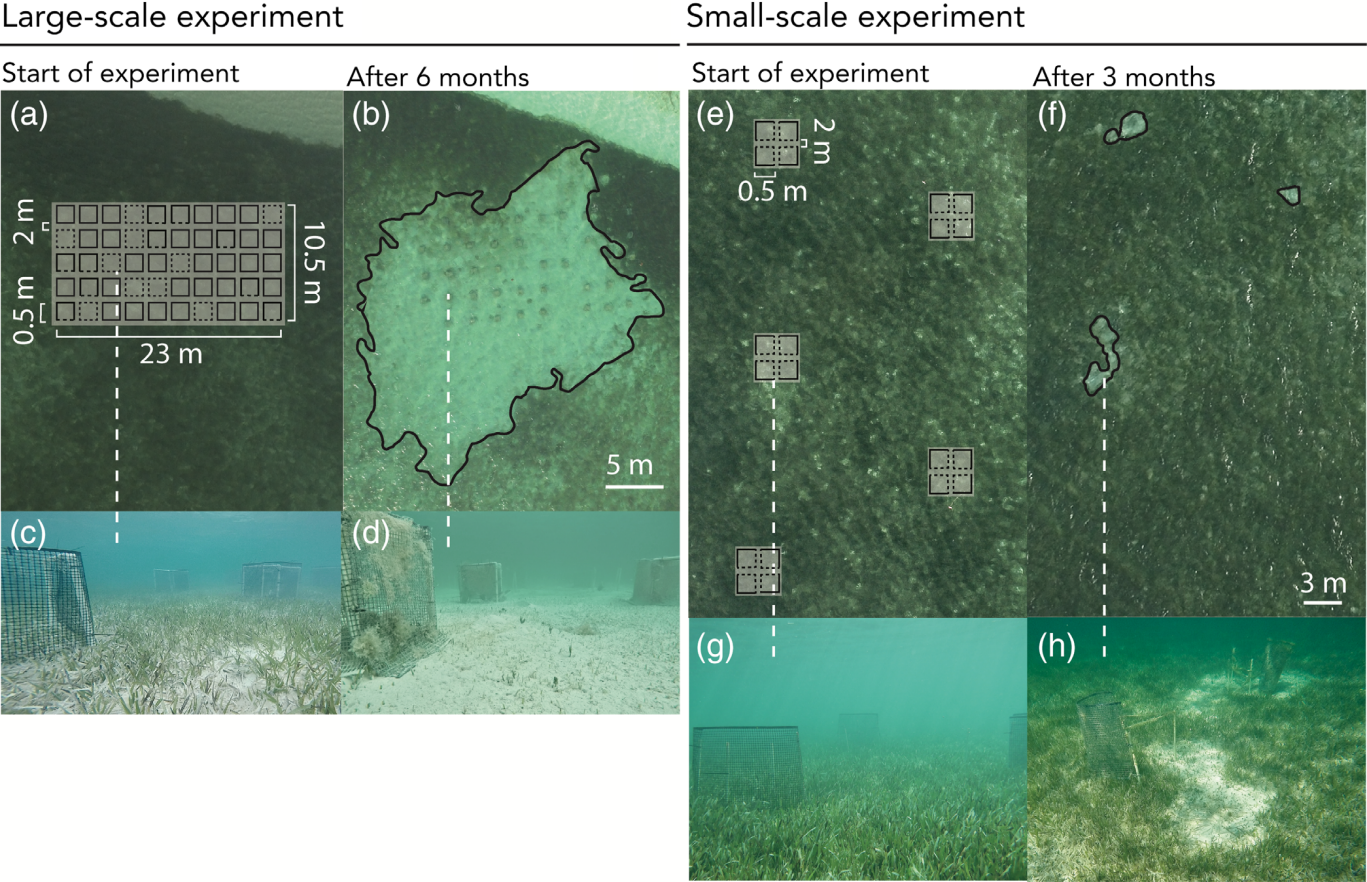
Setup of the experimentally added habitat features and subsequent formation of grazing patches around the large‐scale (a–d) and small‐scale (e–h) arrays. (a, e) Aerial images of the seagrass meadow at the time of structure establishment. The overlay diagrams represent the setup of the artificial structures added as habitat features to the seagrass habitat (details in Appendix S2). (c, g) Corresponding underwater pictures at plot establishment. (b, f) Black outlines of the grazing patches after 6 and 3 months, respectively; (d, h) corresponding underwater pictures. Note the short‐grazed seagrass around the structures in panels (b, d, f, h). Photographs in (c, d, g) accredited to F. O. H. Smulders; photograph in (h) accredited to O. R. O'Shea.

The impact of habitat features on green turtle foraging behavior was tested by establishing arrays of artificial structures at two spatial scales. During the study's initial phase (the large‐scale experiment) we tested the turtle response to the presence of refuges/shelter, represented by a group of artificial structures. An experimental array of (partial) cages, interspersed with open plots, was established as part of a larger experiment studying seagrass herbivory, the *Thalassia* Experimental Network (TEN), led by J. E. Campbell. In total, the setup consisted of a grid of 50 individual 0.5 × 0.5 × 0.5 m (herbivore exclusion) cages and open plots, each separated by 2 m, in an area of 23 × 10.5 m (241.5 m^2^) (see Figure 1a and Appendix S2: Figure S1 for a detailed description). The structures were established on 2 May 2018 and removed 28 March 2019, after 11 months. After removal, four corner poles of the original experimental array were retained to continue measuring the turtle response within the area. At the start of the experiment, a large control area of the same size as the experimental array (23 × 10.5 m) was set up adjacent to the experimental array in an area within a similar continuous seagrass meadow.

Observations of turtle aggregation and grazing in the large‐scale experimental array (Appendix S2: Figure S2), led to a separate follow‐up experiment to study (1) whether this grazing response to the large‐scale array depended on the size of the area with these structures and (2) whether turtles aggregate near the structures to rest inside. Therefore, on 7 November 2019, we established five small‐scale arrays in a similar dense *T. testudinum* meadow approximately 50 m away from the large‐scale experiment, each consisting of four subplots of 0.5 × 0.5 × 0.5 m (9 m^2^ per array), marked with four corner poles, of which two sides were covered with vexar mesh (mesh size 1.5 cm) (Figure 1e). Mesh was not added to the cage tops, preventing turtles from using the structures to rest and sleep in (as was observed in the larger array, Appendix S2: Figure S3).

### Turtle density

We conducted aerial surveys using a drone (DJI Phantom 3) to determine the impact of added habitat features on turtle densities at our study site. The drone was flown to a fixed position 20 m above the large‐scale experimental array and the control area. Both locations were maximum 2.5 m water depth to ensure turtle detection. Perception bias was minimized by only analyzing videos when glare could be minimized to <20% of the field of view, when water clarity allowed easy viewing of the bottom, both in the tall and grazed seagrass, and by viewing the footage three times (following Whitman, 2018, see Video S1 for a turtle moving through both tall and grazed seagrass). For each 10‐min video, the maximum number of turtles observed per given moment was recorded using the MaxN method (Mallet & Pelletier, 2014). Turtles were identified visually and by movement (at least once during the deployments). The aerial surveys were not performed within 5 days after structure addition/removal to minimize impacts of human disturbance on turtle densities. Turtle densities were quantified four times while the large‐scale array was present (October 2018 to February 2019), seven times after the array was removed (in the period of 2–7 months after removal; May to October 2019) and seven times in the control area (October 2018 to October 2019). Turtle densities are expressed as turtles ha^−1^, consistent with previous studies (Christianen et al., 2014; Rodriguez & Heck, 2021).

### Turtle grazing behavior and vigilance

In the large‐scale experiment, we estimated turtle residence time, grazing strategy, and vigilance with and without habitat features using the aerial surveys. Per 10‐min aerial survey, we tracked each individual turtle by labeling it in a video editing program (Wondershare Filmora X10.1.3). In this way, we could quantify the total time (in minutes) each turtle spent within the boundaries of the experimental array. In addition, to describe the behavior for each turtle while inside the array, we calculated the percentage of time each individual turtle spent stationary (grazing/resting in/outside structure), intensively grazing (moving slowly in meandering grazing patterns across the grazing patch), browsing (passing by without intensively grazing and taking occasional bites) and breathing at the water surface. Megaherbivores have been described to decrease both surfacing time (Heithaus & Frid, 2003), and time spent foraging (Wirsing et al., 2007) under the risk of predation. Therefore, in this study we characterized browsing and short surfacing times as high vigilance, while resting and intensively grazing indicated low vigilance. The behavioral characteristics of individual turtles were averaged to obtain a single value per replicate survey.

### Turtle grazing impact

To determine the impact of turtle grazing in between the structures of the large‐scale array on seagrass structure, we measured *T. testudinum* cover, shoot density (No. shoots m^−2^), LAI (Leaf Area Index, one‐sided leaf area m^2^/ground area m^2^), and leaf biomass (g DW m^−2^). The seagrass properties were measured from biomass cores (15 cm diameter) taken within open plots and full cages at the moment of structure removal (11 months after plot establishment, *n* = 4).

The formation of grazing patches was calculated in both the large‐scale and small‐scale experimental arrays by using aerial images made monthly using a drone (DJI Phantom 3). We took underwater images after each drone survey to ground‐truth the grazing patches, confirming that the difference between intensively grazed and ungrazed habitat was indicated by a light green to dark green color border. For the large‐scale experiment, the experimental array was compared with a control area. In ImageJ (ImageJ 1.52q) the grazed area was converted to m^2^ with plot size as a scale reference, assuming a homogeneously flat seafloor.

### Data analysis

All data were tested for normality and homogeneity of variances (Shapiro–Wilk test, Levene test, *p >* 0.05). The difference in average turtle density, turtle residency time and grazing strategy between the large‐scale experiment with structures present versus after structure removal as well as the difference in turtle density between the large‐scale experiment with structures present and the control array were analyzed using Welch two‐sample *t*‐tests (comparing groups with unequal sample sizes and/or variances) or the Wilcoxon rank‐sum test as the nonparametric alternative. The differences in seagrass cover, shoot density, LAI and aboveground biomass between open plots and caged plots were analyzed using two‐sample *t*‐tests (Student *t*‐test for equal variances and Welch *t*‐test for unequal variances), or the Wilcoxon rank‐sum test as the nonparametric alternative. Nonparametric tests were performed on the data with nonnormal asymmetric distributions, because of our small sample sizes. All statistical analyses were performed in R (R Core Team, 2019), *p* < 0.05. Average values are presented together with standard errors.

## RESULTS

### Turtle density

**FIGURE 2:**
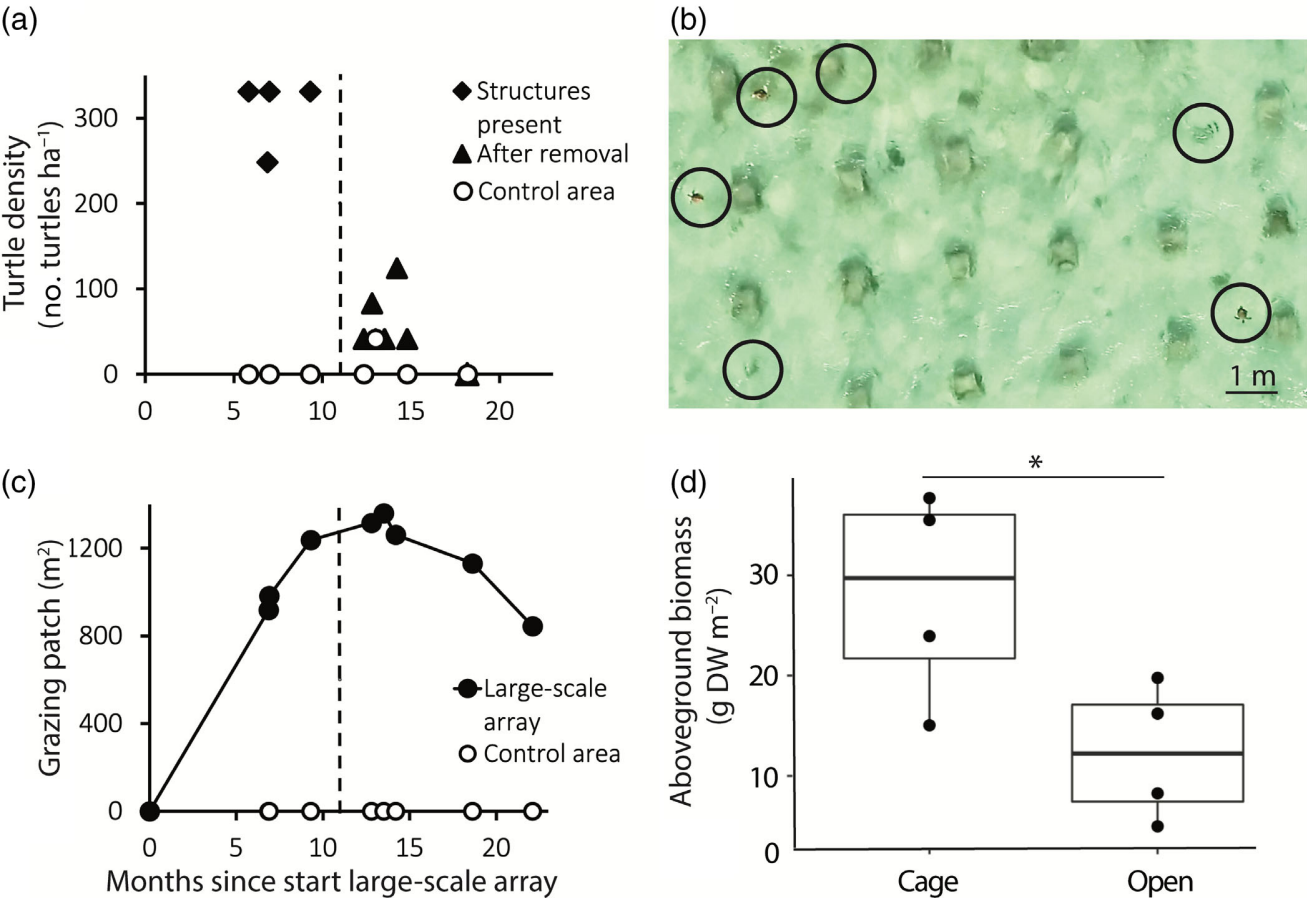
The impact of the large‐scale experimental array with structures on turtle density (a, b), grazing patch development over time (c), and seagrass aboveground biomass (d). (a) Turtle density (MaxN) over time as measured by the aerial surveys in the control area and in the array before and after structure removal. Structure removal, 11 months after establishment, is indicated by the black dashed line. (b) Screenshot of an aerial survey when structures were present, with six turtles present in the frame, of which three are breathing at the surface. The other three could be recognized because they moved in the video. All seagrass surrounding the structures is heavily grazed. (c) Development of the grazing patch surrounding the experimental array since the establishment of the structures (day 0). The black solid line with closed circles indicates the grazed area in the experimental array. Open circles indicate the grazed area in the control area. Structure removal is indicated by the black dashed line. (d) Difference in dry weight (DW) of aboveground *Thalassia testudinum* seagrass biomass between open plots within the array and caged plots at the moment of structure removal (two‐sample *t*‐test, *t*(6) = 2.50, *p* = 0.046). Significant differences between the treatments are indicated with asterisks (*p < 0.05, **p < 0.01, ***p < 0.001).

The average turtle density in the large‐scale array with structures (310.6 ± 20.7 turtles ha^−1^; Figure 2; Video S2) was significantly higher compared with the control area (5.9 ± 5.9 turtles ha^−1^, Wilcoxon rank‐sum test, *W* = 28, *p* = 0.0049), and compared with the large‐scale array 2–7 months after structure removal (53.2 ± 14.9 turtles ha^−1^, Wilcoxon rank‐sum test, *W* = 28, *p* = 0.0084). There was a clear step‐function decline in turtle density after removing the structures (Figure 2a).

### Turtle grazing behavior and vigilance

Turtles varied in their residence time and grazing behavior between the treatments. In the control area only one turtle visited the area during the observations (0.1 min residence time), prohibiting inclusion of the treatment in statistical analysis. Turtles stayed significantly longer in the array with structures present (7.6 ± 0.5 min, *N* = 4) compared with the turtles in the array after structure removal (2.6 ± 0.9 min, *N* = 6, Welch two‐sample *t*‐test, *t*(7.28) = −4.90, *p* = 0.0016). Turtles spent significantly more time stationary (grazing or resting within the seagrass meadow) in the array with structures present (51% ± 5%) compared with turtles in the array after structure removal (6% ± 4%, Wilcoxon rank‐sum test, *W* = 24, *p* = 0.013). Further metrics on behavior either indicating vigilance (browsing, short surfacing times) or nonvigilance (intensive grazing, long surfacing times) are listed in Appendix S4: Table S1.

### Turtle grazing impact

The differences in *T. testudinum* seagrass cover, shoot density, LAI and leaf biomass were compared between open and caged plots situated within the large‐scale experimental array 11 months after the array was established. All seagrass properties were significantly reduced in the open plots compared with the caged plots (Figure 2d; Appendix S3: Figure S1 and Table S1).

Grazing patches were formed both in the large‐scale array, as well as in the follow‐up small‐scale arrays. In the large‐scale array, a single grazing patch of 918 m^2^, covering the area of the experimental plots and beyond (348% of the total array) formed around the structures after 7 months, while no grazing patch was formed in the control area (Figure 1a–d). This patch increased in size to 1359 m^2^ (515% of the size of the total array), until the structures were removed after 11 months (Figure 2c). After structure removal, the patch decreased in area to 319% (844 m^2^), 22 months since the start of the experiment. In the follow‐up small‐scale experiment, turtle grazing patches started to form within 3 months in three out of five replicate arrays (Figure 1e–h; Appendix S3: Figure S2). At 6 months after establishment of the structures, on average 14.4% ± 7.6% (1.3 ± 0.7 m^2^) of the small‐scale experimental arrays were transformed into a grazing patch.

## DISCUSSION

Our study experimentally demonstrates that habitat features can increase green turtle grazing impact, resulting in grazing patches surrounding these features and resultant seascape heterogeneity. Turtles were attracted to both the large‐scale and small‐scale experimental arrays with structures and displayed an increase in nonvigilant behavior as resting and intensive grazing between the structures. Our results suggest that habitat features may serve to reduce the risk of predation for megaherbivores.

### Impact of habitat features on turtle density and grazing behavior

Habitat features, in this case represented by artificial structures, may play an overlooked role in determining foraging site selection and grazing behavior by marine megaherbivores. We found relatively low turtle density and no grazing patches in the control area. In contrast, adding artificial structures to the seascape in a large‐scale array caused a significant local increase in turtle density and their residence time, a significant change of grazing strategy, and the formation of grazing patches, similar to the grazing halos caused by small herbivores (DiFiore et al., 2019). Turtles spent more time resting and grazing within the array compared with the control area and to the array after structure removal. Densities of 331 turtles ha^−1^ within the large‐scale array exceeded previous reports of 18–26 turtles ha^−1^ in high‐density areas, reaching the carrying capacity of those meadows (Rodriguez & Heck, 2021). Indeed, in our study, high turtle density led to a significant decrease in seagrass cover, shoot density, LAI, and aboveground biomass in open plots compared with caged plots. Although herbivore group size by itself could also impact vigilance of individuals and their grazing rates (Bauman et al., 2021), turtle densities were reduced and the grazing patch decreased in size once the structures were removed from the large‐scale array. Moreover, in the follow‐up experiment using small‐scale arrays that supported fewer turtles, individual turtles initiated similar—but smaller—grazing patches in the majority of the arrays, suggesting that the main cause of this change in grazing impact was due to the presence of the structures (see Video S3 in which a single turtle directly targets two of the small‐scale arrays). Multiple mechanisms may be behind this observed effect of habitat features. Below we discuss the main factors that have been found to affect turtle behavior in relation to habitat features, including predation risk, buoyancy regulation, and carapace cleaning.

### Habitat features used as predator refuge may mediate grazing impact in a landscape of fear

Habitat features may reduce the predation risk for turtles, in line with other prey with chasing predators (Creel et al., 2005; DiFiore et al., 2019). In contrast with many sites around the globe where turtles live in predator‐free environments, our study site was situated in a region with high densities of tiger sharks, the main turtle predator (Talwar et al., 2020; Whitman, 2018). Predators can affect prey behavior by creating spatial variation in perceived predation risk through strong nonconsumptive effects, forming a landscape of fear (Gaynor et al., 2019; Laundré et al., 2001). Spatial variation in risk can have larger impacts on prey behavior than direct consumption, as shown for tiger sharks and bottlenose dolphins (Heithaus & Dill, 2002). We observed nonvigilant grazing behavior near the habitat features, and vigilant behavior increased once structures were removed, suggesting an impact of structures on the risk perception of turtles. Although artificial, and of different material, the structures used here had similar dimensions as coral boulders, which often occur in tropical seagrass meadows and are known to provide protection for turtles at night on coral reefs (Christiansen et al., 2017). Our results suggest that these type of habitat features also provide refuge from predators during the day, impacting grazing behavior and thereby shaping the seascape.

How habitat features impact predation risk remains to be investigated. For sharks, which need linear routes of attack (Heithaus, Dill, et al., 2002), vertical structures within open habitats may prevent high‐speed attacks. Alternatively, natural or man‐made structures might deter sharks via other yet unknown mechanisms. For turtles, structures may serve as camouflage to reduce their chances of being visually detected by sharks (Ryan et al., 2022) or limit their need to be vigilant in all directions, as they may be protected from at least one side by the structure, within an otherwise high‐risk homogeneous seagrass meadow. To further investigate these complex predator–prey dynamics, future studies can focus on shark movements and hunting strategy as well as turtle risk perception, orientation, and escape behavior in relation to habitat features. Additionally, it is yet unknown whether this type of risk‐related behavior is intrinsically incorporated in turtle behavior, or if it is linked to local predator presence, which can be clarified with follow‐up studies on the response of turtles to habitat features in low‐predator environments. The presence of habitat features may add a new component important in risk‐related foraging behavior of turtles in addition to body condition, as turtles in poor health have been found to select riskier foraging areas compared with healthy turtles (Heithaus et al., 2007). For future studies we recommend using high‐resolution tracking and animal‐borne video to determine the impacts of both body condition and structures on turtle movements, risk perception, and grazing behavior (Christiansen et al., 2017; Hays et al., 2021; Smulders et al., 2021).

Apart from predation risk, other factors could explain the observed turtle behavior near the structures. The turtles may have used them to regulate their buoyancy while they rest during the day, close to their foraging ground. Because green turtles in water up to 5 m are mostly positively buoyant (Hays et al., 2004), the partial cages may have facilitated resting at this shallow site. Additionally, the structures may have provided a substrate for the cleaning of their carapace (Heithaus, McLash, et al., 2002). However, in the small‐scale experiment, turtles were still attracted to the artificial structures, whereas they could not use these for resting due to the design. Similarly, on the drone videos, mainly grazing and resting was observed, and not cleaning behavior. Therefore, we propose that the structures were likely to be used for foraging site selection in a landscape of fear.

### Implications for future research and nature management

Our findings have implications for other studies on marine grazing behavior. Previously described grazing halos adjacent to natural coral reef structure may partly originate from megaherbivore grazing behavior in addition to mesoherbivores such as fish and urchins (DiFiore et al., 2019) and other (a)biotic processes (Bilodeau et al., 2021). If so, then field studies using a diverse array of (partial) cages and open plots to quantify grazing pressure risk may overestimate local grazing intensity due to the structure effect.

Marine megaherbivores in other (high‐risk) areas are likely to increase their density and grazing pressure near (artificial) habitat features. Permanently added structures to a seagrass habitat may therefore cause a decrease and even loss of seagrass habitat. Conversely, natural resource managers may incorporate artificial structures and/or shelters into their conservation efforts to temporarily concentrate endangered turtle populations in certain areas. Natural structures such as coral boulders may promote local heterogeneity in seagrass structure and therefore ensure a diverse seascape. Our approach and findings provide a novel mechanism that links habitat features and the impact of large marine grazers on the seascape.

## CONFLICT OF INTEREST

All authors affirm that they have no conflicts of interest to declare.

## Supporting information


Appendix S1

Appendix S2

Appendix S3

Appendix S4

Video S1

Video S1 Metadata

Video S2

Video S2 Metadata

Video S3

Video S3 Metadata

## Data Availability

Data (Smulders et al., 2022) are available on the data repository 4TU.Researchdata at https://doi.org/10.4121/20438607.
