# Supplementary material for: Green turtles shape the seascape through grazing patch formation around habitat features: Experimental evidence
Source: Ecology. 2022 Dec 21;104(2):e3902. doi: 10.1002/ecy.3902 (PMC10078154; doi:10.1002/ecy.3902)
Supplement: Supplementary file 1 — Appendix S1 [file ECY-104-0-s010.pdf]

Supporting Information. F.O.H. Smulders, E. S. Bakker, O.R. O'Shea, J.E. Campbell, O. Rhoades, M.J.A. Christianen. Green turtles shape the seascape through grazing patch formation around habitat features: Experimental evidence. Ecology.

Appendix S1. Abundance of tiger sharks (turtle predators) as reported in the wider region of Eleuthera

Table S1. Presence of tiger sharks from studies with data from Eleuthera and neighboring islands. We searched Google Scholar and Web of Science for the key words 'Tiger shark' or '*G. cuvier'* and 'The Bahamas'. Due to limited data, we incorporated both studies on abundance in the region as well as studies where satellite locations of tagged sharks showed that individuals have visited north Eleuthera at least once. From these results, we presented presence of tiger sharks and abundance (in CPUE) if available.

Study	Location	Method	CPUE
Hammerschlag et al., 2015	North Eleuthera	Tagging	
Lea et al., 2015	North Eleuthera	Tagging	
Brooks et al., 2011	South Eleuthera	BRUVS	0.013
		Longline	0.0023
Talwar et al., 2020	South Eleuthera	Longline	0.005
Gallagher et al., 2021	Great Exuma and New providence	Tagging	
Whitman, 2018	Abaco	BRUVS	0.01-0.1

Brooks, E. J., Sims, D. W., Danylchuk, A. J., & Sloman, K. A. (2013). Seasonal abundance, philopatry and demographic structure of Caribbean reef shark (Carcharhinus perezi) assemblages in the north-east Exuma Sound, The Bahamas. *Marine biology*, *160*(10), 2535-2546.

Hammerschlag, N., Broderick, A. C., Coker, J. W., Coyne, M. S., Dodd, M., Frick, M. G., ... & Hawkes, L. A. (2015). Evaluating the landscape of fear between apex predatory sharks and mobile sea turtles across a large dynamic seascape. *Ecology*, *96*(8), 2117-2126

Lea, J. S., Wetherbee, B. M., Queiroz, N., Burnie, N., Aming, C., Sousa, L. L., ... & Shivji, M. S. (2015). Repeated, long-distance migrations by a philopatric predator targeting highly contrasting ecosystems. *Scientific reports*, *5*(1), 1-11.

Whitman, E. R. 2018. Factors affecting green turtle foraging ecology across multiple spatial scales. FIU Electronic Theses and Dissertations. 3870.

Talwar, B. S., Stein, J. A., Connett, S. M., Liss, S. A., & Brooks, E. J. (2020). Results of a fishery-independent longline survey targeting coastal sharks in the eastern Bahamas between 1979 and 2013. *Fisheries Research*, *230*, 105683.

Gallagher, A. J., Shipley, O. N., van Zinnicq Bergmann, M. P., Brownscombe, J. W., Dahlgren, C. P., Frisk, M. G., ... & Duarte, C. M. (2021). Spatial connectivity and drivers of shark habitat use within a large marine protected area in the Caribbean, The Bahamas Shark Sanctuary. *Frontiers in Marine Science*, 1223.
