# Supplementary material for: Green turtles shape the seascape through grazing patch formation around habitat features: Experimental evidence
Source: Ecology. 2022 Dec 21;104(2):e3902. doi: 10.1002/ecy.3902 (PMC10078154; doi:10.1002/ecy.3902)
Supplement: Supplementary file 2 — Appendix S2 [file ECY-104-0-s005.pdf]

Supporting Information. F.O.H. Smulders, E. S. Bakker, O.R. O'Shea, J.E. Campbell, O. Rhoades, M.J.A. Christianen. Green turtles shape the seascape through grazing patch formation around habitat features: Experimental evidence. Ecology.

Appendix S2. Set-up of the large-scale experimental array, and underwater pictures of the array at the moment of establishment, as well as 6 months after establishment.

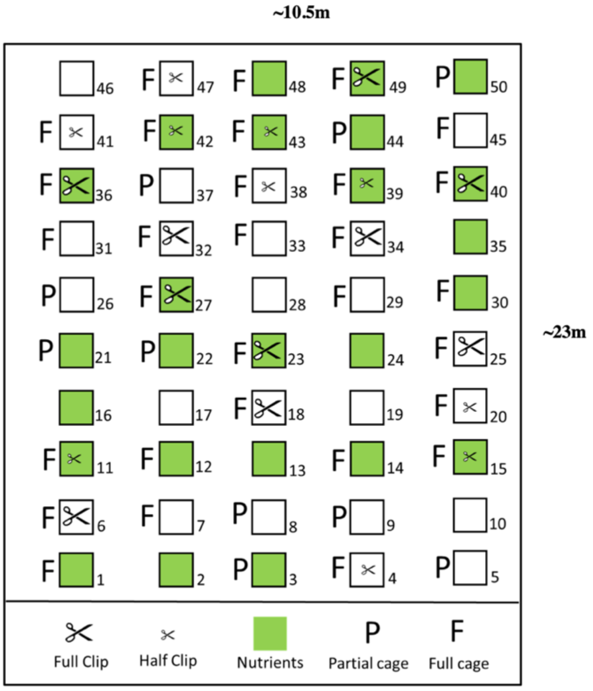

Figure S1. Experimental set-up of the large-scale experimental array, part of the *Thalassia* experimental network (TEN). In total, the set-up consisted of a grid of 50 individual 0.5 x 0.5 x 0.5 m cages and open plots, each separated by 2 m, in an area of 23 x 10.5 m (241.5 m^2). The full cages (n = 30) excluded turtles and consisted of a PVC frame with vexar mesh (mesh size 1.5 cm) on all four sides, and bird mesh on top (mesh size 1.5 cm). Partial cages (n = 10) consisted on the same frame, but with three of the four sides covered in vexar mesh, allowing turtles to occupy the cages. Open plots (n = 10) consisted of four PVC poles marking the base of the frame, without additional poles or mesh on the top or sides. Cage types (and additional treatments, including mimicked grazing and fertilizer addition to plots) were deployed in a fully factorial, randomized design.

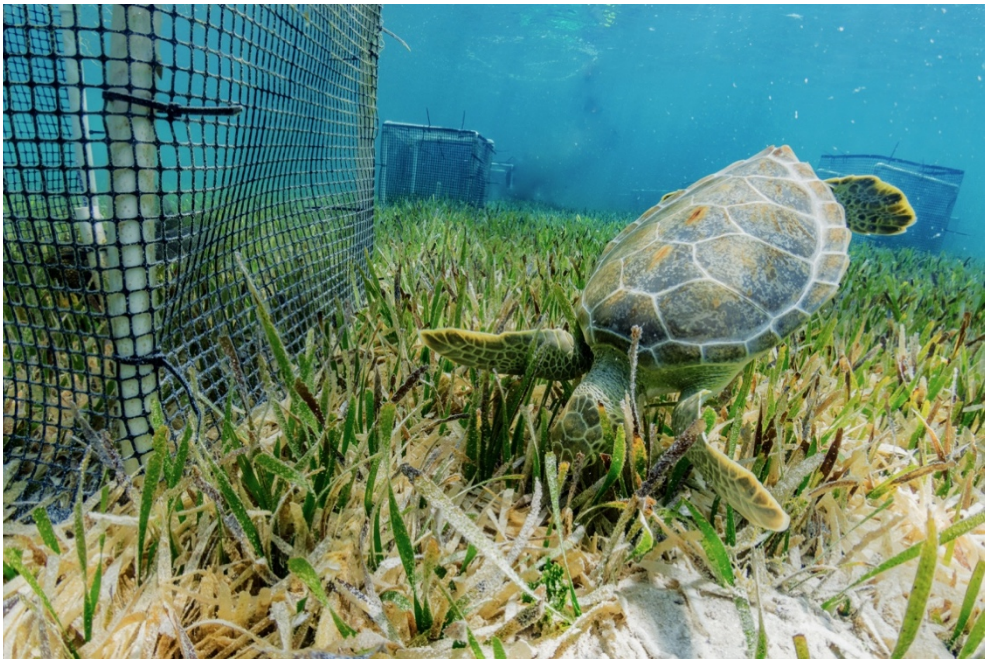

Figure S2. Green turtles increased in density and started grazing near the structures moments after structure establishment, picture taken 2nd of May 2018 by Shane Gross.

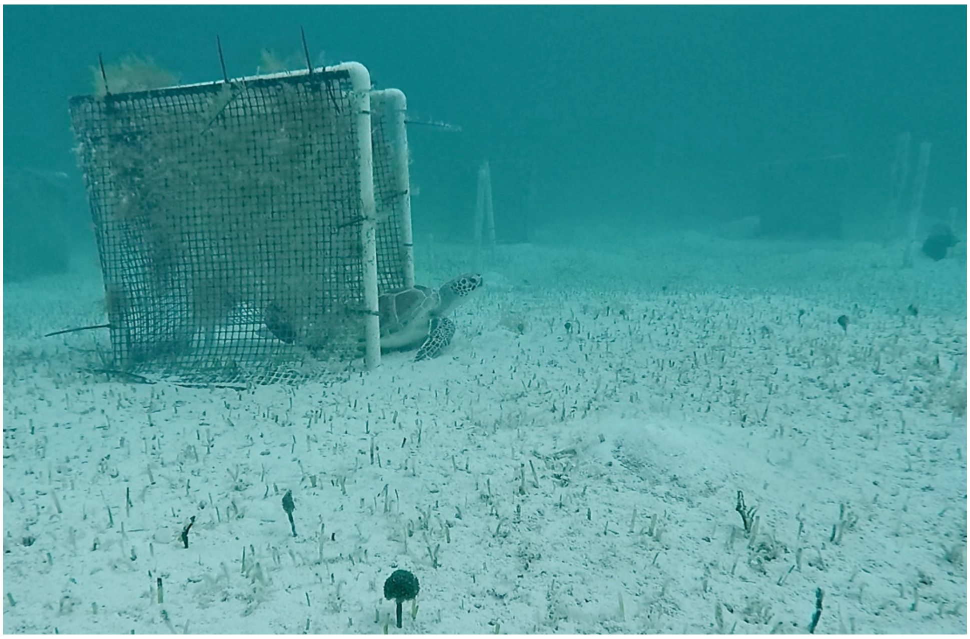

Figure S3. Green turtle in a partial cage of the initial large-scale experiment. Surrounding the partial cage a highly grazed meadow confirms high turtle densities. Picture taken 27th October 2018 by F. Smulders.
