# Supplementary material for: Green turtles shape the seascape through grazing patch formation around habitat features: Experimental evidence
Source: Ecology. 2022 Dec 21;104(2):e3902. doi: 10.1002/ecy.3902 (PMC10078154; doi:10.1002/ecy.3902)
Supplement: Supplementary file 3 — Appendix S3 [file ECY-104-0-s008.pdf]

Supporting Information. F.O.H. Smulders, E. S. Bakker, O.R. O'Shea, J.E. Campbell, O. Rhoades, M.J.A. Christianen. Green turtles shape the seascape through grazing patch formation around habitat features: Experimental evidence. Ecology.

Appendix S3. The impact of artificial structures on seagrass properties and grazing patch formation in the small- and large-scale experiment.

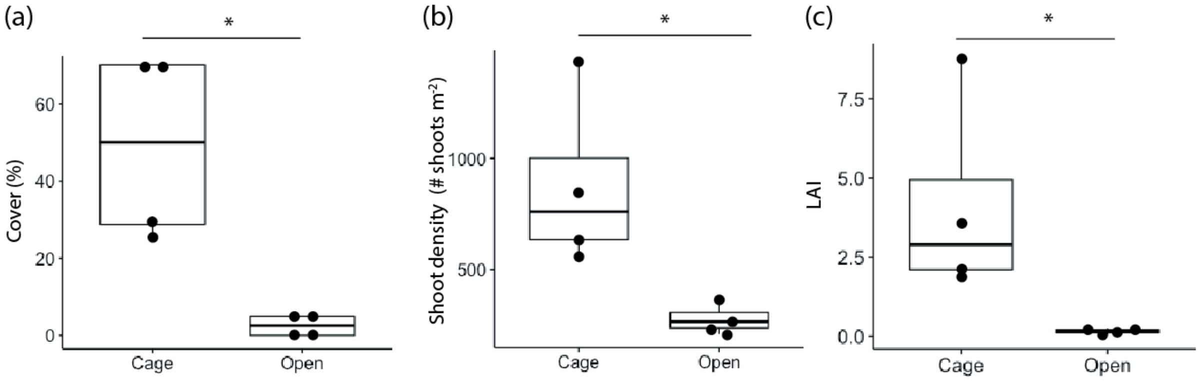

Figure S1. The impact of cage and open treatments the following seagrass properties (a) % cover (Wilcoxon rank-sum test, W = 16, p = 0.027), (b) shoot density (two-sample t-test, t(6) = 3.05, p = 0.023) and (c) Leaf Area Index (LAI) (Wilcoxon rank-sum test, W = 16, p = 0.029), 11 months after the establishment of the large-scale experimental array. Significant differences between cage treatments are indicated with an asterisk (* $p < 0.05$, ** $p < 0.01$, *** $p < 0.001$).

Table S1. *Thalassia testudinum* seagrass properties as measured at the end (11 months) of the large-scale experiment. Values of cover, shoot density, LAI and leaf biomass were compared between the open and caged plot treatments. P values show significant differences between the seagrass properties in the open and caged plots (* $p < 0.05$, ** $p < 0.01$, *** $p < 0.001$).

	Open plots	Caged plots	Test	P-value	Test statistic	df
Cover (%)	2.5 ± 1.4	48.8 ± 12.3	Wilcoxon rank-sum test	**0.030***	W = 16	
Shoot density (shoots m^{-2})	280.7 ± 34.7	876.3 ± 192.3	Student's two-sample t-test	**0.023***	T = 3.05	6
LAI	0.15 ± 0.04	4.14 ± 1.6	Wilcoxon rank-sum test	**0.029***	W = 16	
Leaf biomass (g DW m^{-2})	12.3 ± 3.4	28.1 ± 5.3	Student's two-sample t-test	**0.046***	T = 2.50	6

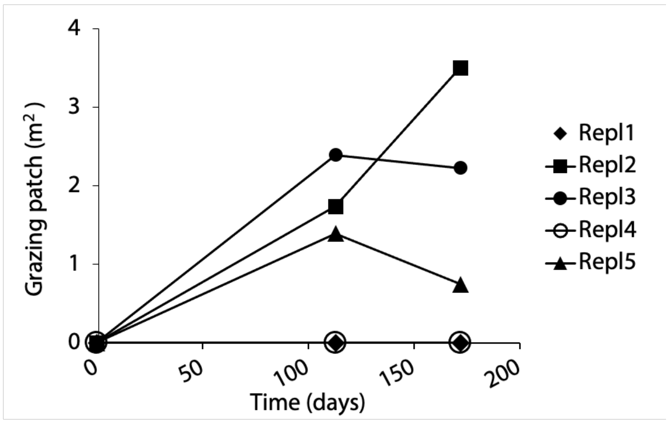

Figure S2. Development of grazing patches surrounding the small-scale experimental arrays (N = 5) since the establishment of the structures (day 0).
