# Supplementary material for: Green turtles shape the seascape through grazing patch formation around habitat features: Experimental evidence
Source: Ecology. 2022 Dec 21;104(2):e3902. doi: 10.1002/ecy.3902 (PMC10078154; doi:10.1002/ecy.3902)
Supplement: Supplementary file 4 — Appendix S4 [file ECY-104-0-s003.pdf]

Supporting Information. F.O.H. Smulders, E. S. Bakker, O.R. O'Shea, J.E. Campbell, O. Rhoades, M.J.A. Christianen. Green turtles shape the seascape through grazing patch formation around habitat features: Experimental evidence. Ecology.

Appendix S4. Turtle behavior in the presence or absence of artificial structures added to their habitat.

Table S1. Behavior and grazing strategy of green turtles in the large-scale experimental array with added artificial structures. Presented is the percentage of time spent within the array (% residence time), stationary (grazing or resting within the seagrass meadow), intensive grazing (moving slowly in meandering patterns across the meadow), intensive grazing + stationary (total grazing), browsing (moving in linear direction) and at the water surface (surfacing). The different treatments consist of turtle behavior in the array while structures were present (N = 4), after structure removal (N = 6) and in the control area (N = 1). P values show significant differences between the turtles in the array with structures present and after removal (* $p < 0.05$, ** $p < 0.01$, *** $p < 0.001$).

	Residency (minutes)	Stationary (%)	Intensive grazing (%)	Total grazing (%)	Browsing (%)	Surfacing (%)
(a) Structures present	7.6 ± 0.5	51.0 ± 4.9	49.0 ± 4.9	100	0	6.3 ± 2.2
(b) After structure removal	2.5 ± 0.9	6.3 ± 4.1	40.2 ± 15.9	46.5 ± 19.0	53.5 ± 17.8	2.5 ± 1.0
(c) Control	0.1	0	0	0	100	0
Test (comparing a & b)	Welch two-sample t-test	Wilcoxon rank-sum test	Welch two-sample t-test	Wilcoxon rank-sum test	Wilcoxon rank-sum test	Welch two-sample t-test
P-value	**0.0016 ****	**0.013 ***	0.62	0.07	0.07	0.18
Test statistic	t = -4.90	W = 24	t = -0.53	W = 20	W = 4	t = -1.60
df	7.28		5.92			4.27
