# Supplementary material for: Green turtles shape the seascape through grazing patch formation around habitat features: Experimental evidence
Source: Ecology. 2022 Dec 21;104(2):e3902. doi: 10.1002/ecy.3902 (PMC10078154; doi:10.1002/ecy.3902)
Supplement: Supplementary file 6 — Video S1 Metadata [file ECY-104-0-s009.pdf]

Video S1: F.O.H. Smulders, E. S. Bakker, O.R. O'Shea, J.E. Campbell, O. Rhoades, M.J.A. Christianen. Green turtles shape the seascape through grazing patch formation around habitat features: Experimental evidence. Ecology.

Caption: Drone video of a green turtle swimming over both the grazing patch and the area surrounding the gazing patch in the large-scale array. This clip illustrates high visibility of turtles both in short in tall-canopy seagrass

Videographer credit: Video S1 was made by Owen R. O'Shea
