# Supplementary material for: Green turtles shape the seascape through grazing patch formation around habitat features: Experimental evidence
Source: Ecology. 2022 Dec 21;104(2):e3902. doi: 10.1002/ecy.3902 (PMC10078154; doi:10.1002/ecy.3902)
Supplement: Supplementary file 8 — Video S2 Metadata [file ECY-104-0-s006.pdf]

Video S2: F.O.H. Smulders, E. S. Bakker, O.R. O'Shea, J.E. Campbell, O. Rhoades, M.J.A. Christianen. Green turtles shape the seascape through grazing patch formation around habitat features: Experimental evidence. Ecology.

Caption: Drone video showing six turtles moving through the large-scale experimental array, individual turtles are encircled with different colours. Structures are visible as dark squares. The light-colored area surrounding the structures consists of the grazed meadow. Darker color in top right of the figure is tall canopy seagrass.

Videographer credit: Video S2 was made by Owen R. O'Shea and edited by Fee O.H. Smulders
